# Binge eating disorder experienced by young doctors struggling with COVID-19

**DOI:** 10.1192/j.eurpsy.2021.765

**Published:** 2021-08-13

**Authors:** M. Tfifha, W. Abbes, M. Dhemaid, K. Mdhaffar, M. Abbes, K. Zitoun, L. Ghanmi

**Affiliations:** Department Of Psychiatry, regional hospital of gabes, gabes, Tunisia

**Keywords:** young doctors, binge eating disorder, COVID19, mental health

## Abstract

**Introduction:**

The COVID19 outbreak has disrupted the mental health of resident doctors who had to care for patients. Eating disorders were among these reported mental health problems.

**Objectives:**

To screen binge eating disorder among young Tunisian doctors and its associated factors.

**Methods:**

We conducted a cross-sectional, descriptive and analytical online-based survey, from April 19, 2020, to May 5, 2020 on 180 medical residents in training. We sent the survey via a google form link. We used a self-administered anonymous questionnaire containing sociodemographic and clinical data of young doctors. The Diagnostic and Statistical Manual of Mental Disorders (DSM-5) criteria were used to assess Binge-Eating Disorder.

**Results:**

Among 180 young doctors who enrolled the survey, 70,2% were female, 16% were married. The mean age was 29 years. 51,1% were frontline caregivers, working directly in diagnosing, treating or caring for patients with coronavirus disease. Among our participants, 5% presented anxiety disorder, another 5 % presented depression disorder and 1,7% had eating disorder. Binge eating disorder were present among 8,9 % of participants and it was associated to personal history of eating disorder (7,7% vs 1,1%, p<10^-3^), past history of depression disorder (7,2% vs 3,3%, p=0.008), exposure to media or news about coronavirus outbreak (0.5% vs 8,3%, p=0.04).

**Conclusions:**

Our study indicated the evolving proportion of binge eating disorder among young doctors. Screening eating disorder is important in order to prevent related physical health problems.

